# Determinants of time to full enteral feeding achievement among infants with birth weight 1000-2000g admitted to the neonatal intensive care unit of public hospitals in Hawassa city, Sidama region Ethiopian, 2019: A retrospective cohort study

**DOI:** 10.1371/journal.pone.0271963

**Published:** 2022-07-28

**Authors:** Melese Tikusie Tewoldie, Meron Girma, Haider Seid

**Affiliations:** 1 Department of Pediatrics and Neonatology, Adare General Hospital, Hawassa, Ethiopia; 2 Food Science and Nutrition Research Directorate, Ethiopian Public Health Institute (EPHI), Addis Ababa, Ethiopia; 3 School of Nutrition, Food Science and Technology, College of Agriculture, Hawassa University, Hawassa, Ethiopia; Sohag University Faculty of Medicine, EGYPT

## Abstract

**Background:**

Nutritional care during the neonatal period is a cornerstone towards achieving optimal care. However, very limited data is available on optimizing parenteral and enteral nutrition that directly affects infant survival among Ethiopian neonates. Therefore, the objective of this study is to identify determinants of time to full enteral feeding achievement among low-birth-weight neonates admitted to neonatal intensive care units of public hospitals in Hawassa city.

**Methods:**

A facility-based retrospective cohort study was conducted in Adare general hospital and Hawassa University’s comprehensive specialized hospital from August 2018 to 2019. Charts of infants with a birth weight of 1000-2000g (n = 273) neonates who were admitted to the neonatal intensive care unit (ICU) were reviewed. The sample size for each hospital was allocated proportionally and subjects were selected by using a simple random sampling technique. Data were entered using Epi. data version 3.1, and analysis was performed using SPSS version 20. Kaplan-Meier estimator and a Cox proportional hazard model were used.

**Result:**

The mean (SD) age when an enteral feed (trophic feeding) was first commenced was 2.13(1.373) days. The median time to achieve full enteral feeding was 8 days with IQR (7–10 days). Gestational age reduces the time to full enteral feeding by 18.8% for each additional week of gestation (AHR = 0.812, p-value = 0.003). The time to achieve full enteral feeding was shorter by 70.4% among neonates who were small for gestational age, as compared with that appropriate for gestational age (AHR = 0.296, p-value<0.001).

**Conclusion:**

According to this study, the time that the neonate takes to achieve full enteral feeding was relatively short. Gestational age and weight for gestational were the determinants for time to full enteral feeding achievement. Further research needs to be conducted to explore further, in addition to current findings.

## Introduction

During the neonatal period, nutritional care is a cornerstone towards providing optimal care to reduce the risk of short-and long-term adverse outcomes [1, 2]. Low birth weight is defined as weight at birth less than 2500 grams. More than 20 million infants are born with low birth weight (LBW) every year and over 96% of them are in developing countries [3]. In those countries, LBW has been a major predictor of child survival [4]. LBW can be caused due to preterm birth (birth before 37 complete weeks of gestation), small size for gestational age (weight for gestation less than 10th percentile), or both [3].

Nutritional care provided to LBW neonates, especially infants with a birth weight of 1000-2000g, is largely either parenteral or enteral nutrition or a combination of both [5]. In most developing countries like Ethiopia, where resources are limited, intravenous fluid instead of parenteral nutrition mainly entails the provision of a maintenance fluid composed of only glucose and electrolyte [6]. The introduction of enteral feeding for those infants will be delayed for several days or longer after birth due to fear of worsening clinical conditions [7]. For many days after delivery, high-risk newborns may stay on maintenance fluid without receiving enteral feeding. This delayed introduction of enteral feeding tends to reduce the gastrointestinal tract’s functional adaptability and increases the need for intravenous fluid, which is risky for different infections and metabolic illnesses [8]. The sequels of a delay in providing adequate postnatal nutrition are significant when they are combined with cumulative deficiencies in protein and energy stores. This results in slower growth, worse neurodevelopmental outcomes, poor bone health, and an increased risk of developing metabolic syndrome later in life [9, 10].

Studies carried out in developed countries show that low birth weight-related morbidity and mortality can be substantially reduced by optimizing parenteral and enteral infant nutrition [2, 6, 11]. However, this is more challenging in developing countries due to differences in low-birth-weight patterns and clinical setup amongst populations. For example, in many of those countries, infants that are cared for are generally greater than 1000 grams. Since it is difficult to establish full enteral feeding faster as much as possible in those whose birth weight is less than 1000grams [6]. This makes the clinician stick with maintenance fluid along with or without a minimal volume of enteral feeding, as the result of the risk of feeding intolerance (FI) and necrotizing enterocolitis (NEC).

Early introduction of enteral nutrition and rapid achievement to full enteral feeding is the ultimate goal for all low-birth-weight neonates in the nutritional care of preterm infants, as it reduces the need for central venous access and thus the risk of infection, and thereby reduces the length of hospital stay [12, 13]. However, in developing countries like Ethiopia, there are very limited parenteral and enteral nutrition practices, despite a high prevalence of infants with a birth weight of 1000-2000g. Thus, to optimize the nutritional management of these infants during the early neonatal period, it is important to set and identify factors associated with the time to full enteral feeding achievement along with different considerations in clinical outcomes. Therefore, this study aims to identify determinants of time to full enteral feeding achievement among infants with a birth weight of 1000-2000g admitted to the neonatal intensive care unit of public hospitals in Hawassa city. The findings of this study are used as evidence to improve enteral feeding practices that are needed to reduce the risk of short-and long-term adverse outcomes of infants with birth weight 1000-2000g.

## Methods and materials

### Study area

The study was conducted in public hospitals in Hawassa city administration, Sidama Ethiopia. Hawassa is the administrative center of SNNPR and Sidama regional state. It is located 275 kilometers south of Addis Ababa. There are 3 public hospitals, 4 non-governmental hospitals, 11 Governmental and 1 non-governmental health center, and 7 diagnostic laboratories in the city. Hawassa university’s comprehensive specialized hospital and Adare general hospital were the two public hospitals that provided the service during the period. The Hawassa University’s comprehensive specialized hospital has a catchment population of 12 million people and it serves about 43,384 patients per year. The Pediatrics department of the hospital has a well-organized neonatal intensive care unit, including personnel and equipment. It provides continuous life support and comprehensive care for extremely high-risk newborn infants and those with complex and critical illnesses. Adare general hospital is not a teaching hospital but provides both preventive and curative services for more than 1,368,341 people. The neonatal ICU is one of the units in the hospital, which gives services under the pediatrics department. The hospital NICU gives all the services, including those with high-risk neonates, except those who need other specialties.

### Study design and period

A facility-based retrospective cohort study design was employed from November 2019 to December 2019.

### Population

#### Source population

All infants with a birth weight of 1000-2000g who were admitted to the neonatal ICU of public hospitals in Hawassa city, Sidama region, Ethiopia.

#### Study population

All randomly selected infants with birth weight 1000–2000 who were admitted to the neonatal ICU of public hospitals during the study period in Hawassa city, Sidama region, Ethiopia.

### Inclusion and exclusion criteria

#### Inclusion criteria

- All LBW newborns between 1000 and 2000 grams who were admitted to NICU within 24 hours of delivery in the selected public hospitals
- All infants with a birth weight of 1000-2000g who were admitted to NICU between August 2018 and 2019

#### Exclusion criteria

- Had major congenital malformations
- Never received enteral feeding because of their clinical conditions (due to GI surgical intervention)
- Died before initiation of enteral feeds
- Records with missing or wrong data on all the predictors

### Sample size determination

The sample size was calculated by using G* power and the following assumption were made while calculating the sample size. The degree of precision or margin of error chosen to be 0.05 with the reliability coefficient of 1.96 certainties (Z = 1.96), 80% power of the study, and 0.2 minimum effect size were taken. Then the final sample size was calculated to be **273**.

### Sampling methods and techniques

**Fig 1:**
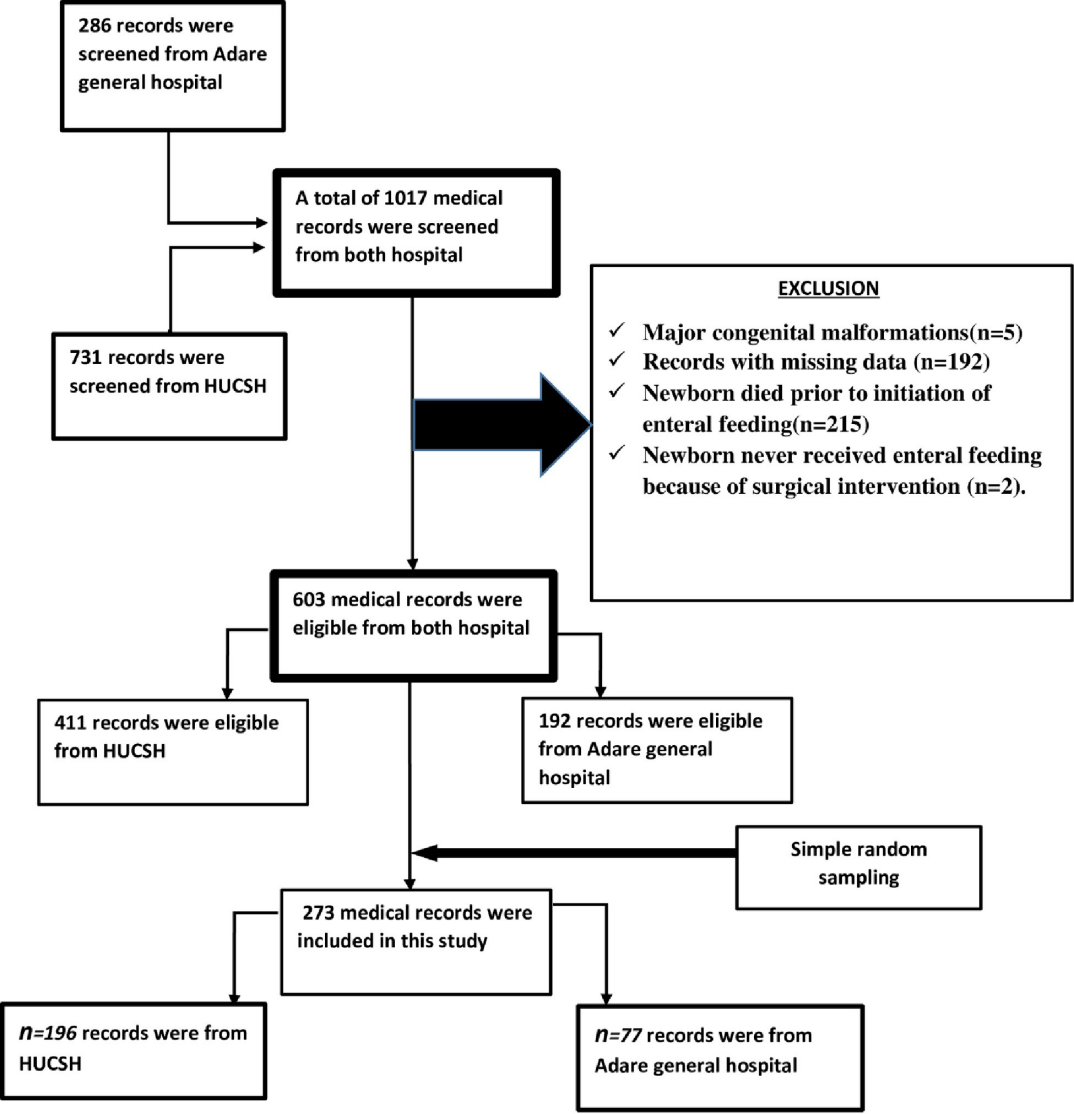
Schematic presentation of the sampling procedure.

Public hospitals that are found in Hawassa city administration, Adare general hospital, and Hawassa university comprehensive specialized hospital (HUCSH) were included in the study because of the service they had in NICU during the specified period. Study inclusion criteria were used to select the study subject from the admission registration book of the two hospitals. Records from August 2018 to August 2019 were reviewed. The sample size was proportionally allocated for the two hospitals depending on the caseload assessed from the registration book for the year before this study. Simple random sampling was employed to draw the final sample size from the two hospitals as it permits a high degree of accuracy due to the limited area of operations of the neonatal ICU “**Fig 1**”.

### Operational definition

Lost to follow-up: subjects drop out of a study for a reason unrelated to the study, such as infants moving or referred from the NICU, leaving against medical advice, and disappearing without leaving an address during the survival period

Incomplete card: when a variable could not be registered or missed on neonatal ICU feeding chart, internal referral paper, and infant medical card

Survival time: Time in day that subjects (infants) spent since the start of trophic until full enteral feeding achieved

Censored: Where subjects died after enteral feeding started, lost to follow-up (drop out), and failed to achieve full enteral feeding before 14 days of follow-up

Event (Failure): The time when infants with a birth weight of 1000-2000g neonates reached full enteral feeding

### Data collection procedures

A retrospective cohort enrollment was completed for infants who were admitted to NICU. The study follow-up period was 14 days. This time is considered the average time when low birth-weight infants started to regain their birth weight, and it is also considered the time when early full enteral feeding should be achieved [14–16].

The data was collected using a checklist that was developed in English. Subjects were screened from the registration book by using a medical record number. Four NICU nurses who had a BSc degree in nursing and trained in NICU nurse’s training extracted data using the checklist; two medical doctors who have been working in NICU supervised the data collection.

Data collectors were trained for one day about the whole course of data extraction, including familiarizing them with the tool, proper data handling, and organizing collected data. A pre-test was done on 10% of the study sample size in Yirgalem hospital neonatal ICU, which is closer but outside the proposed study area. The pre-test was used to test the clarity and understandability of the checklist.

The variables and their definitions used for this study are listed below:

Early-onset neonatal sepsis: Early-onset infections are acquired before or during delivery from birth to 7 days usually less than 72 hrs and diagnosed with Complete Blood Count with differential. Concern for sepsis if total WBC is abnormal (<5,000 or >20,000) and differential with granulocytes >70%.

Antenatal steroid: Antenatal corticosteroids (at least 24–48 hrs. before delivery) given to pregnant women < 34 weeks of gestational period. A complete course of antenatal steroids is diagnosed if a full dose (4 doses of dexamethasone 6mg and 2 doses of betamethasone 12mg) is taken and if a single dose of these drugs is missed, this refers to incomplete.

Gestational age: was estimated by using the last normal menstrual period (LNMP) if the mother can recall her last cycle. If not, an ultrasound supported by Ballard scores is used to assess the physical and neuromuscular maturity of the newborn shortly after birth. This helps to make consistent decisions on gestational age.

Weight for gestational age (WFGA): was determined based on the International Newborn Size Standard charts and categorized the newborns as small for gestational age (SGA) whose weight for gestational age less than the 10^th^ percentile, appropriate for gestational age (AGA) those who are between the 10^th^ and 90^th^ percentile and large for gestational age (LGA) those greater than 90th percentile.

Maintenance fluid: Immediately after birth, following admission, intravenous fluid (80–100 ml/kg/day) composed of 10% dextrose and water started as parenteral nutrition. This has to be advanced by 20 ml/kg/day for the next consecutive days depending on the hydration status of the infant. After the second day of life, other electrolytes like sodium chloride are considered in addition to glucose.

Enteral nutrition: After 24–48 hours of postnatal age, enteral feeding started bilaterally with intravenous fluid as trophic. A small amount of expressed human breast milk or formula milk- 10-20ml/kg/day with a frequency of 3 to 4 times a day is introduced to stimulate and enhance gut function. Daily evaluation of feeding status done by a senior physician every morning and decision made on the way feeding progresses (progressive feeding). This includes escalating the feed by 10–20 ml/kg/day for the date, holding the feed, or keeping it at nothing per os (NPO). As the level of tolerance of the gut increases the need for intravenous fluid becomes less important. This results in reaching full enteral feeding, where intravenous fluid is discontinued and keep the feeding at the volume of 140–150 ml/kg/day or calories of 110–120 kcal/kg/day.

Feeding intolerance (FI): is diagnosed when infants develop either one or more of the following abdominal conditions: vomiting more than 3 times in 24 hours, gastric residual more than 50% before the next feeding, altered aspiration on NGT or OGT (bloody, green, bilious, etc.) and abdominal distension with visible bowel (abdominal girth>2 cm since the last assessment).

Necrotizing enterocolitis (NEC): this is considered if feeding intolerance progress in a worsened form and this may be supported by the unstable vital sign plus or minus abnormal abdominal radiography.

### Data processing and analysis

The data was checked from the checklist for completeness and entered Epi. data version 3.1. The data was then exported to SPSS (Statistical Package for Social Science) version 20 for analysis. Data were cleaned, recorded, categorized, and sorted to facilitate the analysis. Demographic, obstetrics, and clinical characteristics of the study subjects were summarized using descriptive statistics. The Kaplan-Meier estimator was used to calculate the median time to achieve full enteral feeding. Variance inflation factor (VIF) <10 and tolerance statistics > 0.1, were used to conduct a Collinearity diagnostic test to check for the existence of multi-collinearity among the explanatory variables.

A bivariate cox-proportional hazard regression model was used to analyze the association of factors with the time at which full enteral feeding was achieved. Variables that were associated with time to full enteral feeding at 0.25 significant levels in the bivariate model were included in the final multivariable model [17]. Multivariable Cox-proportional hazard models were fitted to assess the association of the time at achieving full enteral feeding with factors. Adjusted hazard ratios with their 95% Confidence Interval (CI) were estimated and P-value less than 0.05 was used to declare the presence of a significant association. Log (-log (st)) plot was used to check the proportional hazard model assumptions.

### Ethics approval and consent to participate

The ethical approval letter, with a reference number of IRB/270/12, was obtained from the Institutional Review Board (IRB) of the College of Medicine and Health Sciences, Hawassa University. The research involves minimal risk, as the review of subjects’ medical records is for limited information. Moreover, the information is not sensitive, and the data are derived from routine, clinically indicated procedures. The need for informed consent was waived or not required as we were doing a retrospective cohort study, and official permission was obtained from HUCSH and Adare general hospital ethical issue concerned bodies. Information that was collected was kept confidential through coding and omitting personal identification. The data were used for research purposes only. The study was conducted per the World Medical Association Declaration of Helsinki-Ethical Principles for Medical Research Involving Human Subjects.

## Results

### Characteristics of study participants

Of the 1017 medical records, (n = 5) were excluded due to major congenital malformations, (n = 192) due to records with missing data, (n = 215) due to death before initiation of enteral feeding, (n = 2) due to newborns never received enteral feeding because of surgical intervention. Among the eligible (603) medical records, 273 of them were extracted for the final analysis.

### Maternal characteristics

Almost all (93.8%) of the mothers of infants were married; 39.6% of mothers were aged 21–25 years, and 49.1% were null parity. Six percent (5.5%) of the mothers did not have any ANC follow-up for the current pregnancy. More than half (55.3%) of the neonates were born by cesarean section (CS) and 19.8% of pregnancies resulted in multiple pregnancy outcomes. Among mothers of LBW infants, 50.2% of mothers had pregnancy-induced hypertension, and 26.4% of them had severe pre-eclampsia. About 40.3% of the mothers who were expecting preterm delivery received antenatal steroid prophylaxis. Among those who received antenatal steroid prophylaxis, only 23.1% of them completed the full course of the treatment. Furthermore, 12.5% of the mothers had a history of prolonged rupture of the membrane. However, only 3.2% of them developed complications and had chorioamnionitis “**Table 1**”.

**Table 1:** Maternal demographic and obstetric characteristics of LBW neonates admitted to the neonatal ICU in Hawassa city, Sidama region, Ethiopia, 2019.

Variables (n = 273)		Frequency	%
**Marital**	**Status**		
	Married	256	93.8
	Unmarried	14	5.1
	Separated	3	1.1
**Age of the mother (year)**			
	< = 20	44	16.1
	21–25	108	39.6
	26–30	81	29.7
	31–35	30	10.9
	> = 36	10	3.7
**Parity (for current pregnancy)**			
	Null parity	134	49.1
	Parity > or = 1	139	50.9
**ANC follow up**			
	Yes	258	94.5
	No	15	5.5
**Pregnancy-induced hypertension (PIH)**			
	No	136	49.8
	Yes	137	50.2
**Degree of PIH**			
	Mild-pre-eclampsia	36	13.1
	Sever-pre-eclampsia	72	26.3
	Eclampsia	29	10.6
**Antenatal Steroid prophylaxis**			
	Complete	63	23.1
	Incomplete	47	17.2
	Never received	163	59.7
**Prolonged rupture of membrane**			
	Yes	34	12.5
	No	239	87.5
**Chorioamnionitis**			
Yes		8.8	3.2
No		264	96.8
**Mode of delivery**			
	CS	151	55.3
	SVD	120	44
	Assisted vaginal delivery	2	0.7
**Multiple pregnancies**			
	Single	219	80.2
	Multiple	54	19.8

### Neonatal characteristics

**Fig 2:**
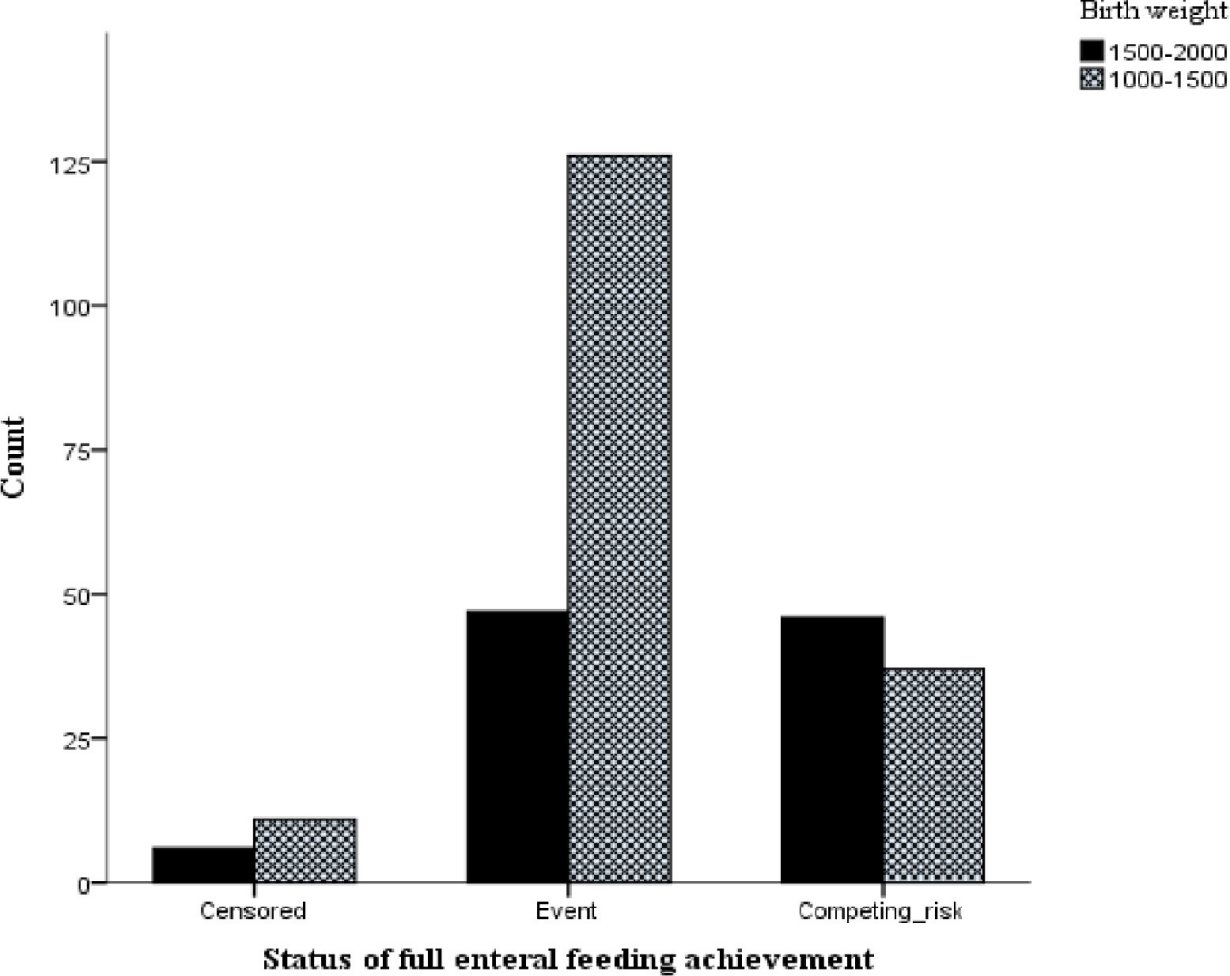
Status of full enteral feeding achievement with respect to the birth weight of neonates admitted to the neonatal ICU.

As shown in “**Table 2”** out of 273 infants, 55.7% were male. The mean (SD) of gestational age (GA) and birth weight was 33.8 (1.3) weeks and 1578 (245.9) grams, respectively. Infants with a birth weight of 1000-2000g in this cohort, those whose birth weight is between 1000-1500g less likely to achieve full enteral feeding earlier than 1500-2000g with a mean value of 7.31 vs 5.72days, respectively “**Fig 2”.** Among those infants who were included in this study, 20.5% were small for their gestational age (SGA) (less than the 10th percentile). Of the neonates, 30.4% were treated for clinical sepsis (early-onset neonatal sepsis). The Apgar score, determined in the 1st and 5th minutes, was low for 53.1% and 20.5% of infants, respectively. Respiratory distress syndrome was also diagnosed in more than half of low-birth-weight infants (53.2%). Of the participants, 7 (2.6%) and 9 (3.3%) were fed on formula and mixed types of milk, respectively “**Table 2**”.

**Table 2:** Neonatal demographic and clinical characteristics of LBW neonates admitted to the neonatal ICU in Hawassa city, Sidama region Ethiopia, 2019.

Variables (n = 273)		Frequency	%
**Sex of the neonate**			
	Male	152	55.7
	Female	121	44.3
**Birth weight (gm)**			
	1000-<1500	99	36.3
	1500-<2000	174	63.7
**Gestational age at birth (week)**			
	28-<32	15	5.5
	32-<37	258	94.5
**Weight for Gestational age**			
	AGA	217	79.5
	SGA	56	20.5
**Early-onset neonatal sepsis**			
	Yes	83	30.4
	No	190	69.6
**Neonatal hyperbilirubinemia**			
	Yes	100	36.6
	No	173	63.4
**Respiratory disease syndrome**			
	Yes	145	53.2
	No	128	46.8
**APGAR 1**^**st**^ **minute**			
	0–6	145	53.1
	7–10	128	46.9
**APGAR 5**^**th**^ **minute**			
	0–6	56	20.5
	7–10	217	79.5
**Resuscitate at birth**			
	Yes	82	29.8
	No	191	70.2
**Type of feeding**			
	Human breast milk	257	94.1
	Formula milk	7	2.6
	Mixed type	9	3.3

### Follow-up and full enteral feeding attainment patterns of the cohort

Follow-up and full enteral feeding attainment patterns of the cohort, as shown in the figure below, from those who were in this cohort, 100(36.6%) were censored due to: death 83 (30.4%), losses to follow-up 6(2.2%), and not experiencing full enteral feeding attainment before the study end 11(4.02%). Of those who are in this cohort, 30.4% of them were dead after enteral feeding had started this is due to RDS, EONS, low Apgar score, etc. The mean (SD) of age when an enteral feed (trophic feeding) was first commenced was 2.13(1.373) days, and 63.4% of the neonates achieved full enteral feeding within 14 days.

**Fig 3:**
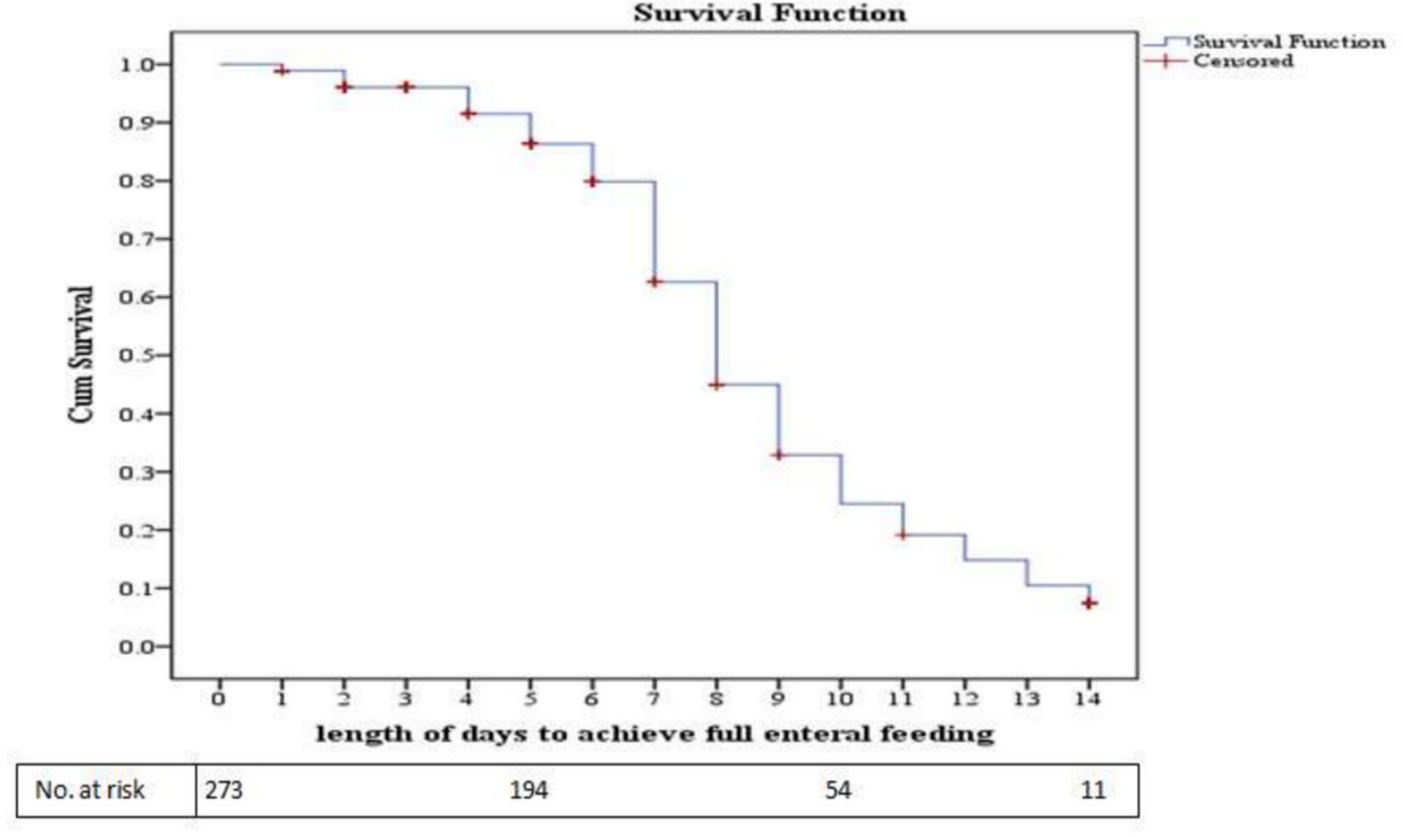
Kaplan-Meier survival function curve of full enteral feeding achievement for neonates admitted to the neonatal ICU.

The median (IQR) time of attainment of full enteral feeding was 8 (7–10) days. The total person-time that the cohort yields in this study were 1,838 person-days (5.04 infant-years) of follow-up. The incidence rate of full enteral feeding achievement was calculated to be 9.4 per 100 person-day. As shown in “**Fig 3”**, about 194 and 54 infants were likely to achieve full enteral feeding achievement on the 5th and 10th days of the follow-up, respectively.

### Determinants of time to full enteral feeding achievement

To determine which variables, determine the full enteral feeding achievement, maternal and neonatal clinical and obstetrics characteristics were assessed for their association with full enteral feeding achievement using cox regression. Variables that passed the bivariate analysis and multi-collinearity test were included in the multivariate analysis. Variables such as sex, marital status, pregnancy-induced hypertension, multiple pregnancies, antenatal steroid prophylaxis, Apgar score (at 1st and 5th min), mode of delivery, birth weight, type of milk, and resuscitation at birth failed to pass bivariate analysis at p-value less than 0.25 **"Table 3”.**

**Table 3:** Bivariate Cox-regression analysis of associated factors of full enteral feeding achievement of LBW neonates admitted to the neonatal ICU in Hawassa city, Sidama region Ethiopia, 2019.

Variables	Frequency	Crude HR	P-value
**Prolonged rupture of membrane (PROM)**			
Yes	34(12.5)	1.375(0.876–2.158)	0.166
No	239(87.5)	Ref	
**Weight for gestational age (WFG)**			
AGA	217(79.5)	3.835(2.497–5.892)	<0.001
SGA	56(20.5)	Ref	
**Respiratory distress syndrome (RDS)**			
Yes	145(53.1)	0.697(0.510–0.953)	0.024
No	128(46.9)	Ref	
**Neonatal hyperbilirubinemia**			
Yes	100(36.6)	1.222(0.900–1.660)	0.199
No	173(63.4)	Ref	
**Early-onset neonatal sepsis (EONS)**			
Yes	83(30.4)	0.713(0.511–0.994)	0.046
No	190(69.6)	Ref	
**Gestational age(week)**	273(100)	0.763(0.664–0.838)	<0.001
**Birth weight (gm)**			
1000-<1500	99(36.3)	0.886(0.626–1.253)	0.492
1500-<2000	174(63.7)	Ref	
**APGAR 1**^**st**^ **minute**			
0–6	145(53.1)	0.89(0.658–1.200)	0.444
7–10	128(46.9)	Ref	
**APGAR 5**^**th**^ **minute**			
0–6	56(20.5)	0.97(0.662–1.435)	0.899
7–10	217(79.5)	Ref	
**Pregnancy-induced hypertension (PIH)**			
No	136(49.8)	0.99(0.734–1.335)	0.947
Yes	137(50.2)		
**Antenatal Steroid prophylaxis**			
Complete	63(23.1)	0.887(0.616–1.277)	0.519
Incomplete	47(17.2)	0.888(0.577–1.368)	0.591
Never received	163(59.7)	Ref	
**Chorioamnionitis**			
Yes	8.8 3.2)	1.152(0.652–2.035)	0.625
No	264(96.8)	Ref	
**Mode of delivery**			
CS	151(55.3)	0.856(0.626–1.170)	0.330
SVD	122(44.7)	Ref	

Whereas gestational age, prolonged rupture of membrane (PROM), weight for gestational age (WGA), early-onset neonatal sepsis (EONS), respiratory distress syndrome (RDS), and neonatal hyperbilirubinemia were included in the final model.

**Fig 4:**
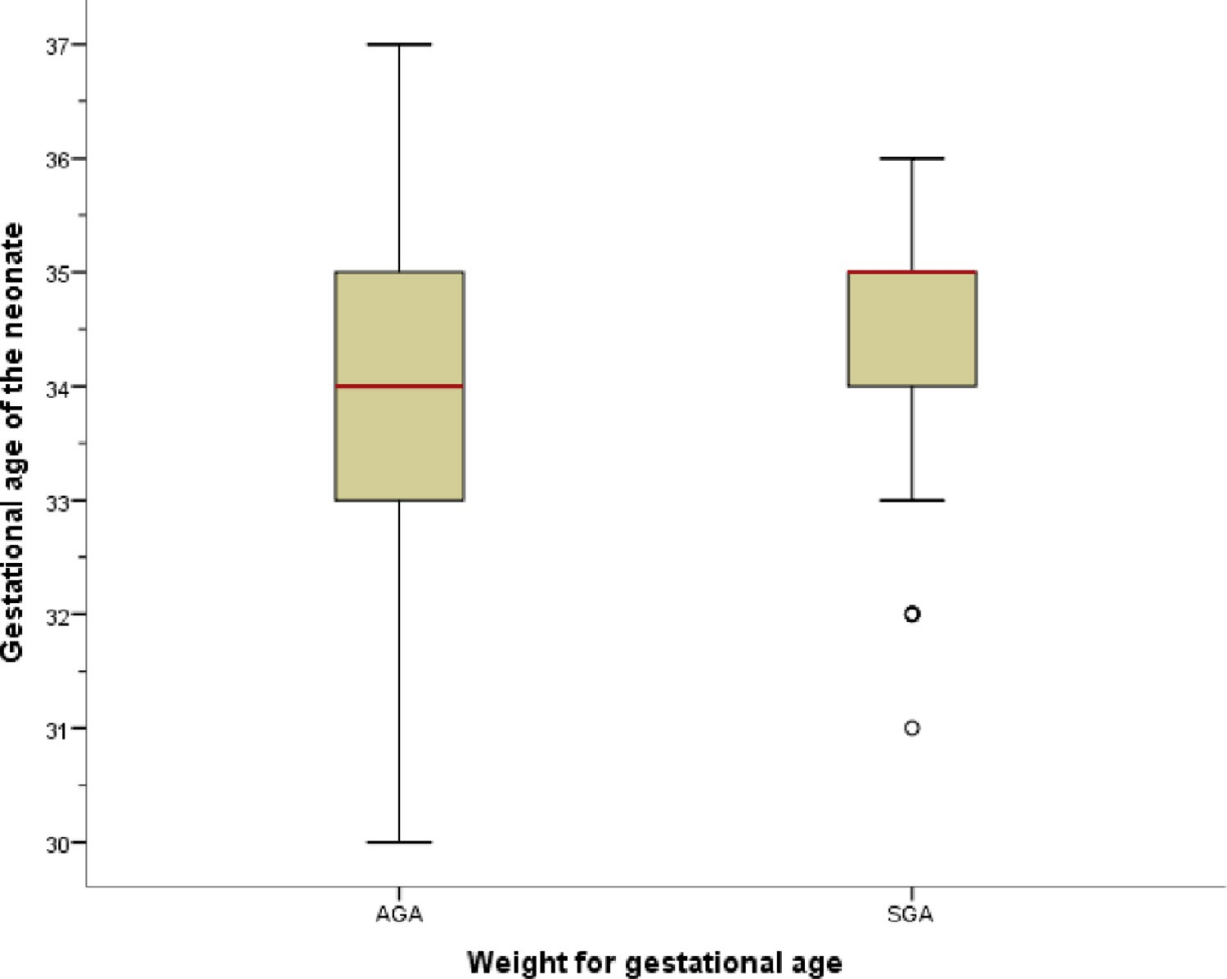
Box plot of birth weight of AGA and SGA neonates admitted to the neonatal ICU.

In the multivariate analysis, only gestational age and weight for gestational age were significantly associated with the time to full enteral feeding achievement. GA and WFGA were negatively associated with the time to full enteral feeding achievement. After adjustment for RDS, PROM, EONS and neonatal hyperbilirubinemia, GA reduces the time to full enteral feeding by 18.8%, GA (AHR = 0.812, 95%CI = (0.708–0.931, p-value = 0.003)). Being small for gestational age (SGA) reduced the time to full enteral feeding achievement by 70.4% as compared to appropriate for gestational age (AGA) (HR = 0.296,95%CI = (0.187–0.467), p-value = <0.001). A subgroup analysis between AGA and SGA shows Test Statistics (TS) = -5.134, and p-value = <0.001 “**Fig 4**”. However, PROM, EONS, RDS, and neonatal hyperbilirubinemia were not statistically significant even if they were included in the last model “**Table 4**”.

**Table 4:** Bivariate and multivariate Cox-regression analysis of associated factors of full enteral feeding achievement of infants with birth weight 1000-2000g admitted to the neonatal ICU.

Variables	Frequency		Crude HR	Adjusted HR
	Censored (%)	Event (%)		
**Prolonged rupture of membrane (PROM)**				
Yes	12(12)	22(12.7)	1.375(0.876–2.158)	1.042(0.624–1.740)
No	88(88)	151(87.3)	Ref	Ref
**Weight for gestational age (WFG)**				
AGA	68(68)	32(32)	3.835(2.497–5.892)	0.296(0.187–0.467) **
SGA	145(83.8)	28(16.2)	Ref	Ref
**Respiratory distress syndrome (RDS)**				
Yes	67(67)	80(46.2)	0.697(0.510–0.953)	0.941(0.674–1.314)
No	33(33)	93(53.8)	Ref	Ref
**Neonatal hyperbilirubinemia**				
Yes	31(31)	69(39.9)	1.222(0.900–1.660)	0.766(0.560–1.049)
No	69(69)	104(60.1)	Ref	Ref
**Early-onset neonatal sepsis (EONS)**				
Yes	30(30)	70(70)	0.713(0.511–0.994)	1.211(0.825–1.775)
No	51(29.5)	122(70.5)	Ref	Ref
**Gestational age (week)**	100	173	0.746(0.664–0.838)	0.812(0.708–0.931) **

HR: hazard ratio; CI: confidence interval

**: p-value <0.01, Log (-log (survival)) plot used to test proportional hazards assumption for covariates

Ref = Reference

## Discussion

The present study explored the time that infants with a birth weight of 1000-2000g spend in NICU to achieve full enteral feeding. This study found that more than sixty percent of infants with a birth weight of 1000-2000g achieved full enteral feeding within 14 days of the follow-up period. They demonstrate a shorter time to achieve full enteral feeding as compared to other studies.

The median time to reach full enteral feeding was 8 days with IQR (7–10 days), and the mean GA was 33.8 weeks. This is much lower than that was reported from Italy but higher from India, 13 days IQR (7–24 days) with a mean GA of 29+1 week and 7 days IQR (7–9.5 days) with a mean GA of 30.8 weeks, respectively [13, 18]. There are also studies whose median time is slightly higher than that of the current findings from Italy, India, and the UK: 11 days IQR (7–20 days) with a mean GA of 29 weeks; 9.5 days with a mean GA of 35.5 weeks; and 11 days IQR (8–13 days) with median GA of 26 weeks, respectively [6, 19, 20]. This may be due to the difference in the mean gestational age between the two populations. The mean gestational age of the population in the current study was relatively higher than in the earlier study in Italy, India, and the UK. However, another multicenter study from UK was in line with the current finding, with a median time to achieve full enteral feeding of 8 days IQR (6–11 days) with the median gestational age of 32+2 weeks, provided that there is gestational age and clinical setup difference between the two populations [21].

As expected, higher gestational age was a predictor for the fastest achievement of full enteral feeding. As GA increases in a week, the time to achieve full enteral feeding decreases by 18.8%. This is slightly lower than a study from Italy, which was 15.5%, even though it is difficult to compare up-front with the current study regardless of issues like patterns of low birth weight and clinical variability [13]. There is evidence that as the gestational age increases, the time needed to reach full enteral feeding becomes shorter. This could be due to a well-developed and matured gastrointestinal system they have. Moreover, a well-developed and matured gastrointestinal tract abides by a minute and progressive volume of milk given as a treatment, which also determines the tolerance level of the feeding [2, 13, 22]. This in turn reduces the time that the neonates spend on trophic feeding and hospital stay, and reaches full enteral feeding earlier than those who are with small GA.

Weight for gestational age (WFGA) was statistically significant, with full enteral feeding achievement. According to the result of this study, being small for gestational age (SGA) is protective- it reduces the time to full enteral feeding by 70.4% more than that of appropriate for gestational age (AGA). As previously mentioned, this may also be due to the gestational age difference in the category that increases gut development and maturation as gestational gets higher. Moreover, this could be due to failure in early initiation of enteral nutrition and advancement to full in infants with birth weight 1000-2000g with small GA, which is important in rapid maturation of the gastrointestinal system and lesserfeeding intolerance.

There are a few similar studies that correlate being small for gestational age (SGA) with GI maturation. According to Patwardhan et al. [2], the time to full enteral feeding achievement was negatively affected by being SGA or not in the group. Furthermore, this study also concludes that SGA shows a reduction in time to full enteral feeding achievement as compared to AGA. This is also due to the difference in mean gestational age in these two groups, and mean GA being 30 weeks was compared with 32.9 weeks maturity in AGA and SGA, respectively. However, subgroup analysis between AGA and SGA shows Test Statistics (TS) = -5.134, and p-value = <0.001.

Mihatsch et al. [23], also show that SGA with abnormal umbilical artery Doppler ultrasound and not SGA has no difference in reaching full enteral feeding. This study tried to compare very-low-birth-weight infants with SGA whose umbilical artery blood flow has resistance on Doppler ultrasound (placental insufficiency) and infants with a normal weight for gestational age. Therefore, more than 65.5% of SGA infants had abnormal doppler or a history of Pregnancy-induced hypertension.

Attempts should have been designed to address limited practices of parenteral and enteral nutrition in infants who are admitted to the NICU. This study finding may be used as evidence to improve enteral feeding practices that are needed to reduce the risk of short and long-term adverse outcomes for infants due to extended periods of hospital stay.

Variable that has possible confounder effects were early-onset neonatal sepsis and respiratory distress syndrome this may be adjusted in a model of multivariate cox regression.

The major limitation of this study is a high percentage of attrition and missing data as a result of being a retrospective study. Furthermore, the study is limited to some clinical variables which need an advanced clinical setup to find out participants’ characteristics.

## Conclusion

According to this study, the time that the neonate takes to achieve full enteral feeding was relatively short. Gestational age and weight for gestational were the determinants for time to full enteral feeding achievement. Further research needs to be conducted to explore further, in addition to current findings.

## Abbreviations

AGA: Appropriate for Gestational Age
CS: Caesarian Section
EBM: Expressed Breast Milk
EONS: Early Onset Neonatal Sepsis
FI: Feeding Intolerance
GA: Gestational Age
HUCSH: Hawassa University Comprehensive Specialized Hospital
LBW: Low Birth Weight
NICU: Neonatal Intensive Care Unit
NEC: Necrotizing Enterocolitis
PIH: Pregnancy Induced Hypertension
SGA: Small for Gestational Age
SNNPR: Southern Nations Nationality Peoples Region
SVD: Spontaneous Vaginal Delivery
VLBW: Very Low Birth Weight
WHO: World Health Organization
